# Supplementary figures and images for: In Silico Repurposed Drugs against Monkeypox Virus
Source: Molecules. 2022 Aug 18;27(16):5277. doi: 10.3390/molecules27165277 (PMC9415168; doi:10.3390/molecules27165277)

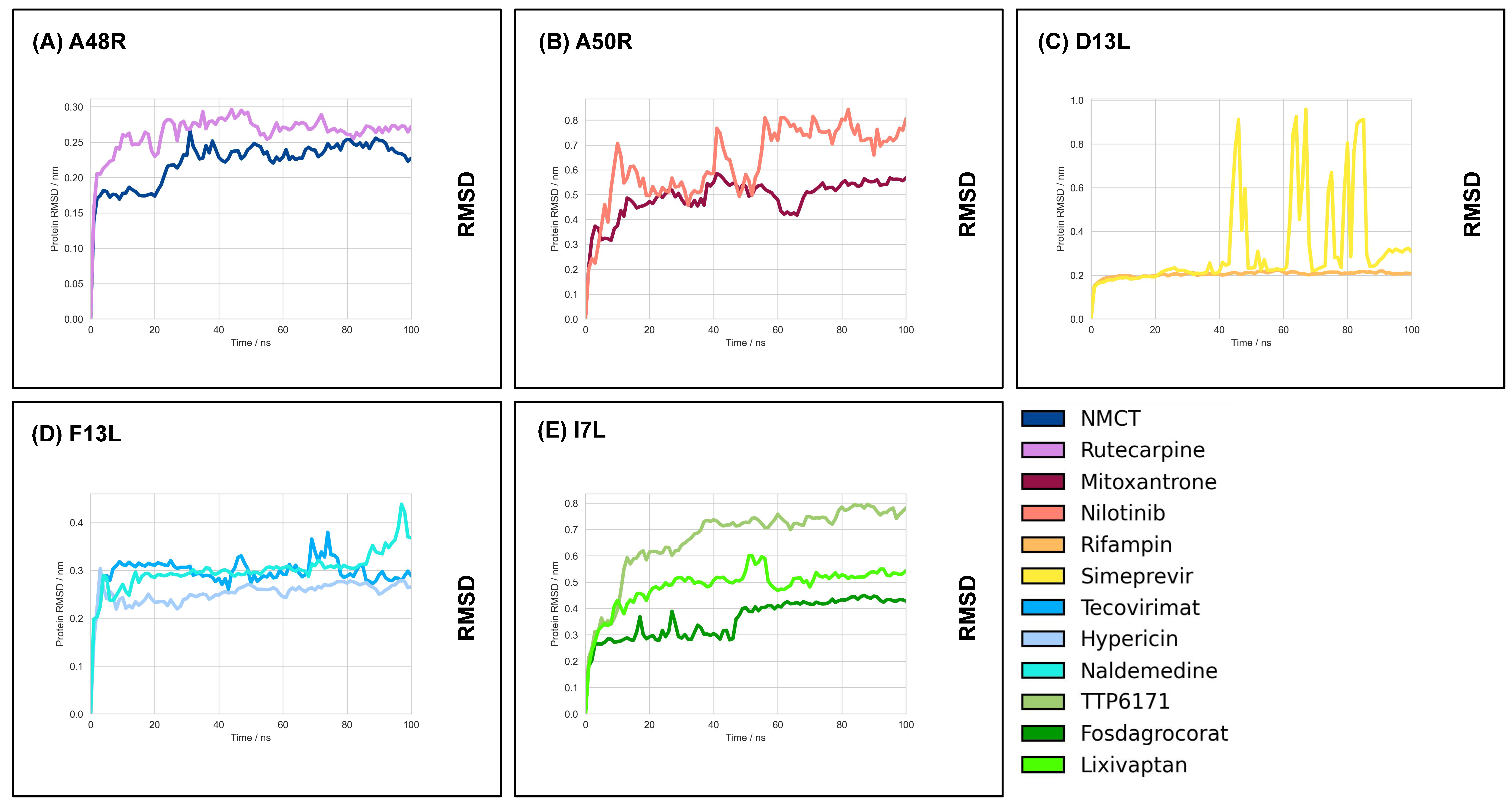

Supplement: Supplementary file 1 [file molecules-27-05277-s001.zip › Supplementary figure S3.png]
